# A Case of Polytrauma in a Farmer Following a Cow Attack: "Code Farmer"

**DOI:** 10.7759/cureus.84497

**Published:** 2025-05-20

**Authors:** Steven J Laxton, Jeffery Cloyd

**Affiliations:** 1 Department of Emergency Medicine, University of Tennessee Health Science Center (UTHSC) Nashville - St. Thomas Health, Nashville, USA; 2 Department of Emergency Medicine, University of Tennessee Health Science Center (UTHSC), Knoxville, USA

**Keywords:** code farmer, contiguous rib fractures, farm animal injuries, farmer, liver laceration, polytrauma, rib fractures, spleen laceration

## Abstract

This case involves a farmer who presented to a level 1 trauma center following an attack by a cow. It highlights the emerging use of the term “code farmer” among emergency department physicians and nurses, a colloquial alert reflecting the concern that farmers may present with seemingly minor complaints that often mask more serious or insidious underlying injuries or health conditions.

The phrase “code farmer" is an emerging term gaining traction among emergency medicine physicians and nurses. It reflects a recognition that farmers often endure strenuous work environments, long hours, and a high pain tolerance, making them less likely to seek medical attention unless faced with injuries or pathologies that demand urgent and serious intervention. Farmers are routinely exposed to significant occupational hazards, including chemical, environmental, mechanical, and livestock-related risks, and chronic health challenges.

This case exemplifies the occupational risks faced by farmers. It describes a patient who sustained polytrauma involving the face, thorax, and abdomen, including multiple solid organ injuries following a cow attack. Despite the severity of his condition, his initial chief complaint was simply shortness of breath. He ultimately required intensive care unit (ICU) admission and multidisciplinary medical and surgical management.

## Introduction

Farming is widely recognized as one of the highest-risk occupations for health hazards, with a mortality rate five times higher than that of all other professions combined. In other words, a farmer is five times more likely to die from a workplace-related accident than the average worker [1]. These occupational hazards are diverse and include mechanical risks (i.e., tractor accidents, entanglement in machinery), chemical exposures (i.e., pesticides, herbicides, acute chemical poisoning, and long-term risks such as carcinogenesis), livestock-related injuries (i.e., being kicked, trampled, or bitten by livestock), environmental challenges (such as working in extreme heat or cold environments, leading to temperature-related illnesses), respiratory hazards (i.e., exposure to dust, mold, and noxious agricultural gases), inadequate safety training and a limited safety culture, and exposure to zoonotic infections [2,3]. The patient in this case sustained multi-organ trauma following a cow attack, ultimately requiring escalation of care to the trauma intensive care unit (ICU). 

Contiguous rib fractures, especially in individuals over the age of 65, are associated with significantly increased mortality due to the complications that can arise from these injuries. Elderly patients are particularly vulnerable to rib fractures owing to age-related bone demineralization and decreased thoracic compliance. Contiguous fractures, defined as the fracture of two or more adjacent ribs, can compromise the structural integrity of the chest wall and severely impair respiratory mechanics. This disruption can precipitate complications such as pneumonia and atelectasis, both of which are linked to increased mortality in this population. Studies have shown that elderly patients with multiple rib fractures are more likely to experience prolonged hospitalizations, increased need for mechanical ventilation, and higher rates of ICU admission, all of which contribute to increased mortality risk [4].

Pain resulting from rib fractures also plays a critical role in adverse outcomes. Inadequate pain control may lead to hypoventilation, impaired cough, and reduced mobility, factors that heighten the risk of pulmonary complications, particularly pneumonia. Additionally, the elderly exhibit a diminished physiological reserve and a less robust response to trauma, further impairing their ability to recover from such injuries [5].

## Case presentation

The patient is a 64-year-old male who presented to the emergency department as a trauma alert via air ambulance after being stomped and crushed by a bull. According to his wife, the incident occurred while they were vaccinating a calf; the mother cow became aggressive, charged at them, knocked over a fence gate onto the patient, and then stomped on the gate. On arrival, the patient reported abdominal and chest pain, as well as pain with breathing. His primary complaint, however, was shortness of breath. Prehospital assessment by emergency medical services (EMS) revealed hypoxia, requiring supplemental oxygen at 6 L per minute via nasal cannula to maintain normoxia. Otherwise, his vital signs were within normal limits, and there was no evidence of hemodynamic instability. During the primary trauma survey, the patient was found to have an intact airway and diminished breath sounds on the left side, without obvious signs of circulatory compromise. Due to the presence of distracting injuries and an inability to clear the cervical spine clinically, a cervical collar (C collar) was applied as a precaution.

The secondary survey revealed a Glasgow Coma Scale (GCS) score of 15. The patient exhibited facial bruising with associated mid-face and jaw pain. Chest examination demonstrated palpable crepitus along the anterior and left chest, with significant tenderness to palpation across the chest. Abdominal assessment revealed bruising in the lower quadrants and generalized tenderness without focal peritoneal signs. The patient had diffuse abrasions throughout the chest, abdomen, and extremities. A focused assessment with sonography for trauma (FAST) exam was not performed initially, as the patient maintained stable blood pressure and was deemed appropriate for an immediate CT scan. Although breath sounds were diminished on the left side, chest tube placement was deferred until arrival in the trauma ICU, as there was no clinical or radiologic evidence of tension pneumothorax or hemodynamic compromise at the time.

**Figure 1:**
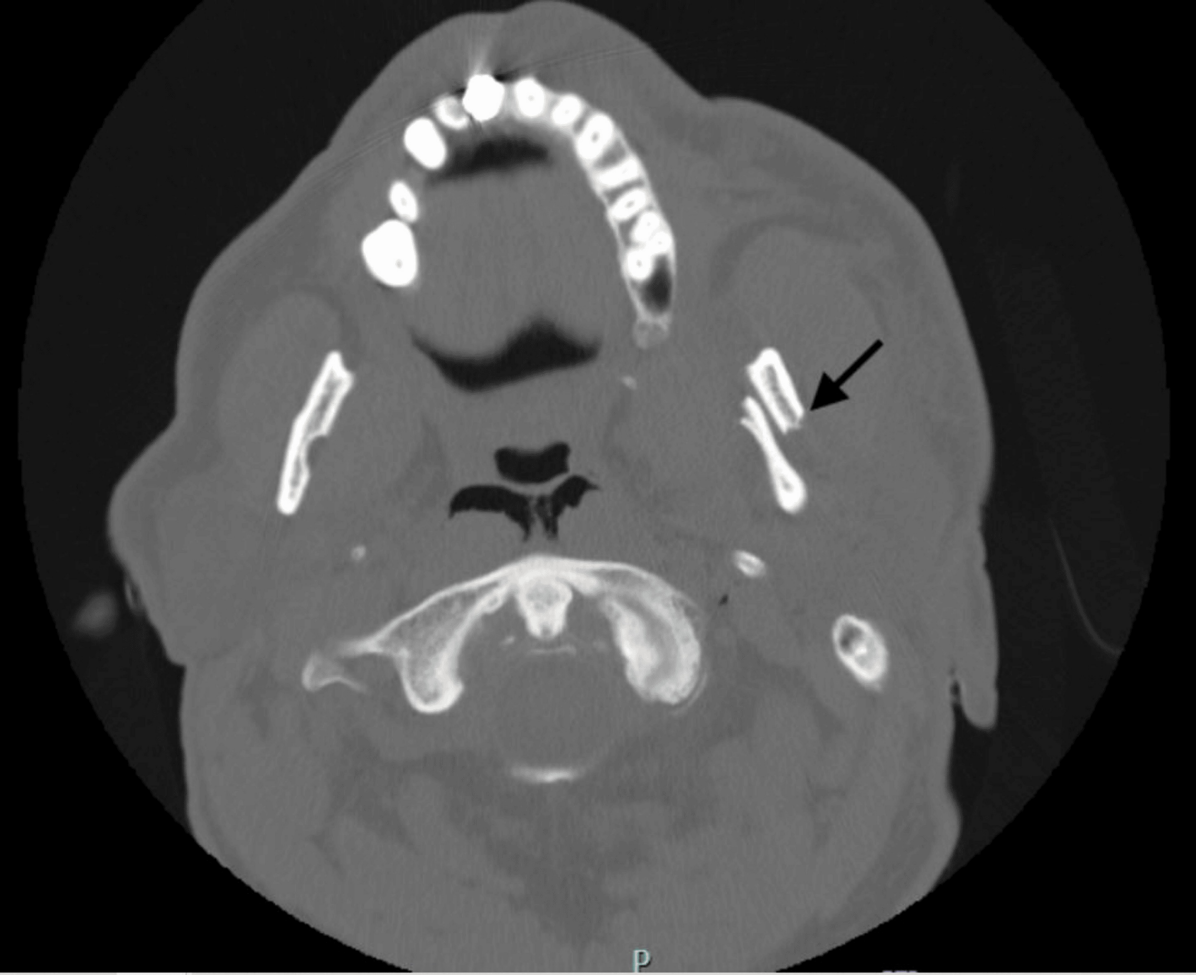
CT scan of the face showing a displaced left mandibular fracture (black arrow)

**Figure 2:**
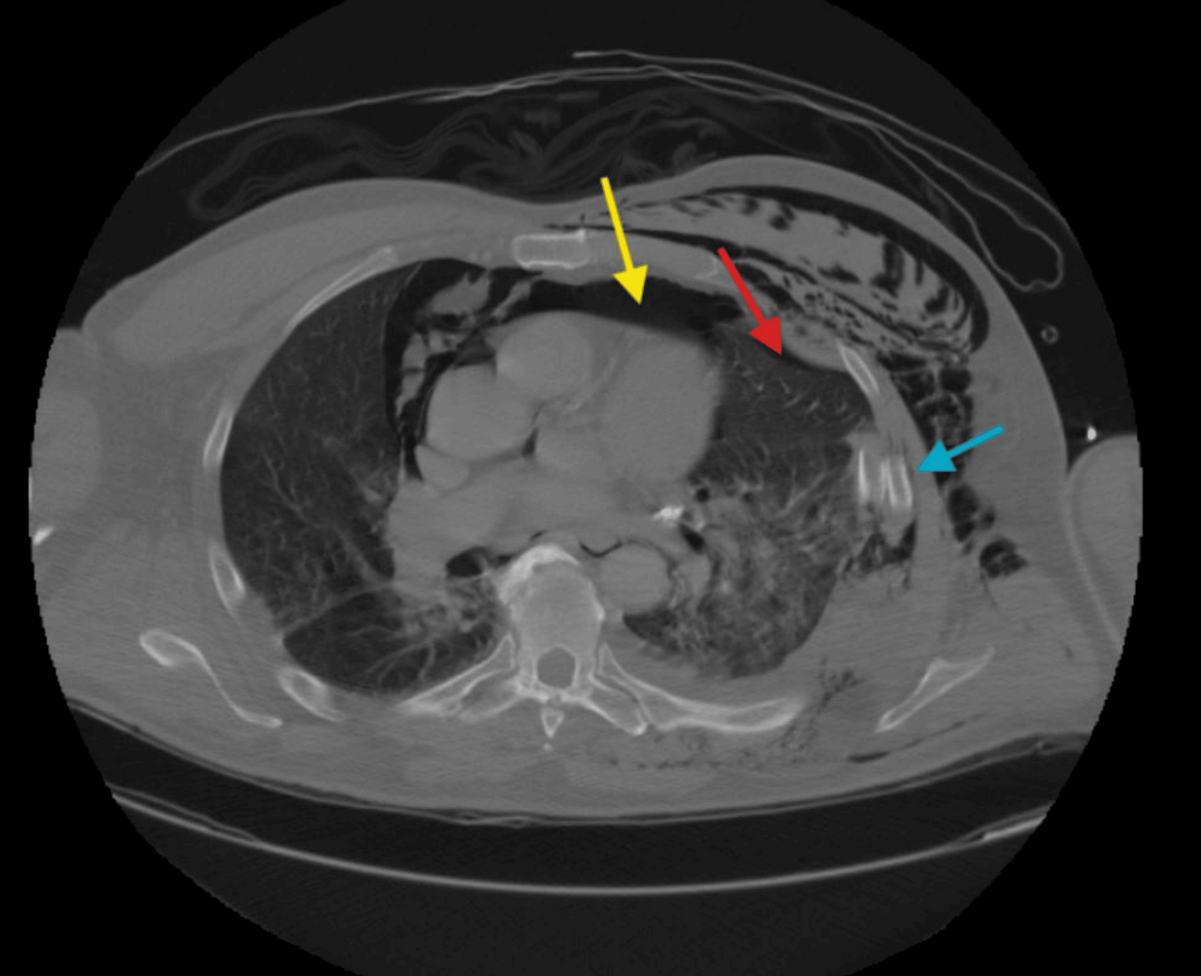
CT scan of the thorax revealing a left pneumothorax (red arrow), pneumomediastinum (yellow arrow), and multiple displaced rib fractures (blue arrow)

**Figure 3:**
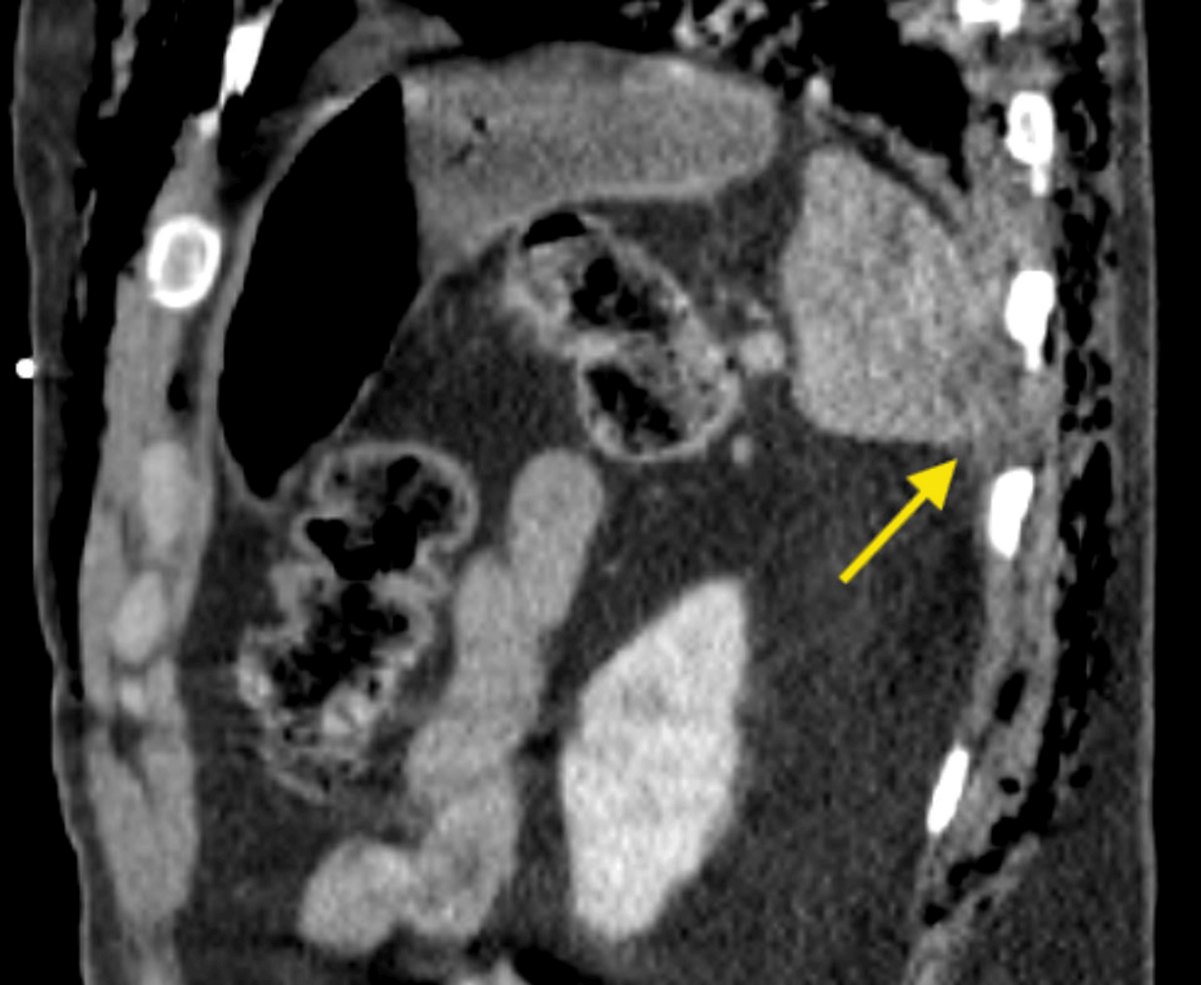
CT scan of the abdomen showing a splenic laceration with associated intra-abdominal hematoma (yellow arrow)

**Figure 4:**
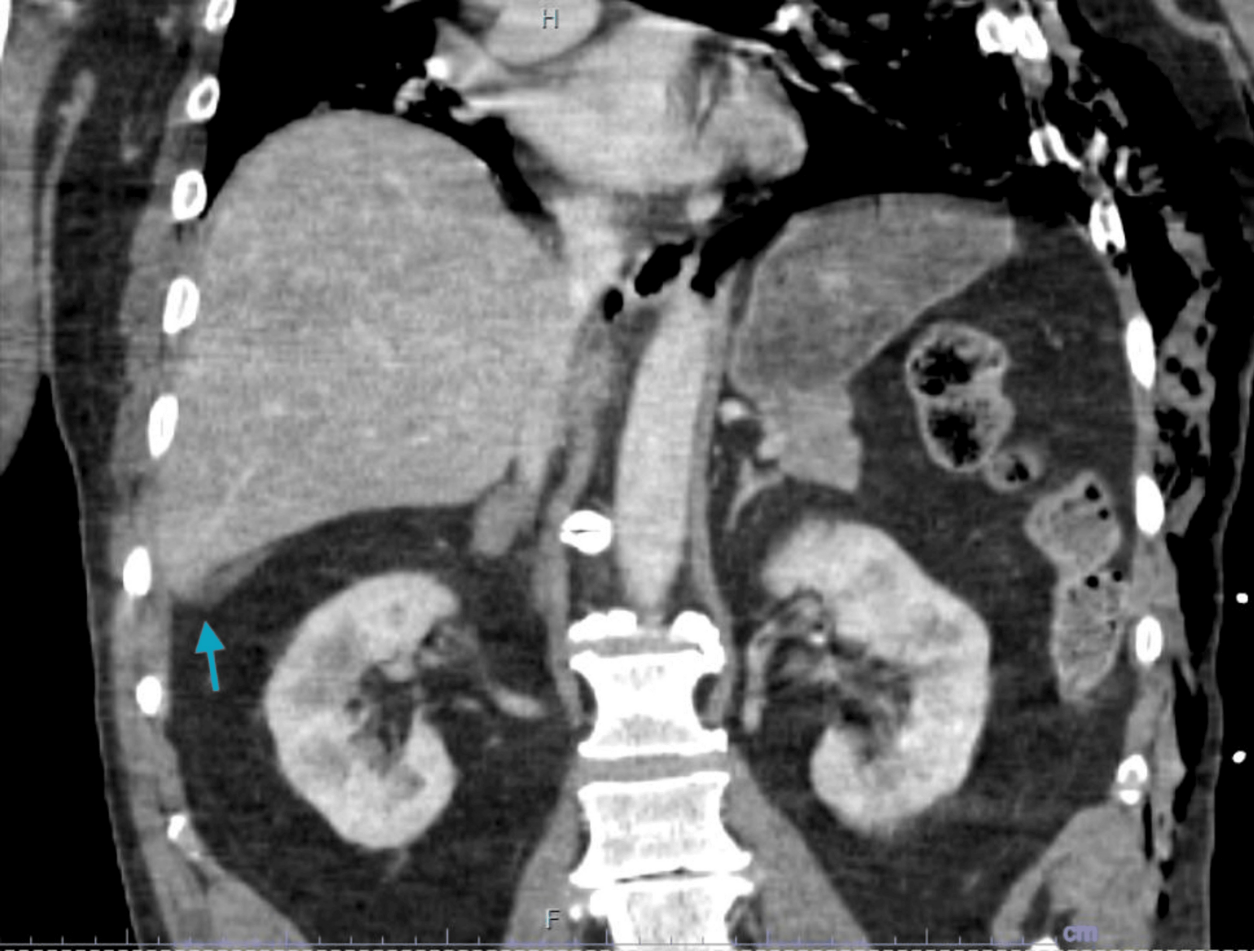
CT scan of the abdomen demonstrating a grade 3 laceration to the right hepatic lobe with surrounding peri-hepatic hematoma (blue arrow)

The patient underwent comprehensive trauma imaging, including non-contrast CT scans of the head, maxillofacial region, and cervical spine, as well as contrast-enhanced CT scans of the chest, abdomen, and pelvis, which revealed multiple traumatic injuries. Notably, he sustained a left mandibular fracture (Figure 1), a fracture of the left posterolateral wall of the maxillary sinus, and a fracture of the left lateral pterygoid plate. Thoracic injuries included left-sided rib fractures (ribs 2-9) with significant displacement, a left pneumothorax, pneumomediastinum, and right-sided rib fractures (ribs 6-10) (Figure 2). Abdominal findings included a 3.3 cm right adrenal hematoma, trace peri-splenic fluid consistent with a spleen laceration (Figure 3), and a grade 3 liver laceration (Figure 4). Additional injuries included T5-T6 left transverse process fractures and a T1 anterior superior corner avulsion fracture. Bilateral pulmonary contusions were also noted. 

The patient's emergency department course was otherwise unremarkable, without further decompensation prior to hospital admission. 

He was subsequently admitted to the trauma ICU for continued monitoring and management of his multiple injuries. He was treated with multimodal pain control; however, despite these measures, the patient demonstrated poor progress with incentive spirometry, attributed to significant pain from the markedly displaced rib fractures. The anesthesia pain management team was consulted and initiated a continuous intravenous lidocaine infusion at 2 mg/kg/h, which provided partial relief. Nonetheless, due to persistent respiratory limitations and inadequate pain control, surgical intervention was ultimately required for rib fracture stabilization.

The patient's intra-abdominal injuries, including liver and spleen lacerations, were managed conservatively without the need for surgical intervention.

For facial trauma, the oral and maxillofacial surgery (OMFS) team was consulted regarding the left mandibular ramus fracture. The patient underwent open reduction and internal fixation (ORIF) with plate placement. The procedure was completed without complications, and the patient experienced an uneventful postoperative recovery.

The patient's extensive thoracic trauma was initially managed with the insertion of a 28 Fr chest tube in the left anterolateral chest. Despite aggressive multimodal pain management, including a lidocaine infusion, he continued to experience chest pain that impaired effective breathing, required ongoing supplemental oxygen, and resulted in low incentive spirometry volumes. Due to persistent symptoms and the severity of displacement, surgical fixation was performed, with plating of the left-sided rib fractures at ribs 5, 6, and 7; rib 7 required plating at two separate locations.

Postoperatively, the patient showed progressive improvement in respiratory function and pain control. He was subsequently discharged to a rehabilitation facility for further recovery before eventual discharge home.

## Discussion

Healthcare providers have long recognized that farmers face unique occupational health risks that can lead to serious, sometimes insidious conditions, with delayed medical presentations being common [6]. This awareness has prompted emergency physicians and nurses to adopt the term “code farmer,” reflecting the suspicion that a farmer’s seemingly straightforward complaint may mask a severe underlying condition. The term draws a parallel to other critical hospital codes, such as “code blue” or “code stroke.” The patient in this case initially only called EMS for shortness of breath following a cow attack but was subsequently found to have multiple traumatic injuries affecting several organ systems, each significantly increasing his risk of morbidity and mortality.

Occupational hazards faced by farmers, including exposure to pesticides, operation of heavy machinery, interaction with livestock, contact with high-tension wiring, and physically demanding labor, contribute to a wide range of both acute injuries and chronic health conditions [7]. 

A study of farmers in Georgia, USA, identified key barriers to effective healthcare engagement, including time constraints related to their demanding work schedules, rising healthcare costs, and a perceived lack of cultural competence among providers in appreciating the unique risks and exposures inherent to farming [8]. These challenges frequently contribute to the underutilization of healthcare services, allowing subtle health issues to remain undetected until they advance to more severe stages.

Additionally, prior literature involving rural farmers showed concerns about access to quality healthcare, with participants emphasizing the importance of healthcare services that are accessible, flexible, and culturally sensitive. The study also noted that farmers' deep commitment to their work often leads them to prioritize farm operations over personal health, potentially delaying the recognition and treatment of emerging health issues [9].

These insights underscore the need for healthcare providers to be vigilant and proactive in assessing the health of farming populations, considering that both occupational risks and sociocultural factors may obscure early signs of serious health conditions.

Prior studies have shown that elderly patients who sustain rib fractures are at increased risk of long-term complications as well. These include functional decline, chronic pain, and impaired quality of life. The combination of acute respiratory distress and chronic health issues exacerbates the risk of mortality in this population. Older patients with rib fractures have a significantly higher mortality rate compared to younger adults, with mortality rates escalating for those with severe fractures or multiple contiguous rib fractures [10]. These findings emphasize the importance of early intervention, appropriate pain management, and vigilant monitoring for potential respiratory and cardiovascular complications in elderly patients who sustain rib fractures. In this case, the patient required rib plating due to the severe pain he experienced and significant displacement of rib fractures. 

In addition to the multiple contiguous rib fractures, this patient also experienced multisystem trauma. In individuals over the age of 65, multisystem trauma significantly increases the risk of mortality due to the compounded effects of multiple organ injuries and the aging body’s reduced ability to recover from trauma. The elderly population often have a reduced physiological reserve, meaning that their bodies are less able to handle the stresses of trauma. As people age, they experience a decline in organ function, immune response, and muscle mass, all of which contribute to poorer outcomes in the face of severe injuries. When trauma affects multiple organ systems, such as the cardiovascular, respiratory, renal, and neurological systems, the body’s ability to compensate and recover becomes severely compromised. Elderly adults with multisystem trauma had significantly higher mortality rates compared to younger patients, primarily due to the cumulative burden placed on their diminished physiological systems [11].

The risk of mortality is also further elevated in elderly trauma patients due to the higher likelihood of pre-existing comorbidities, which often complicate the management of multiple organ system injuries. Conditions such as hypertension, diabetes, cardiovascular disease, and chronic obstructive pulmonary disease are common among the elderly and can exacerbate the effects of trauma. For instance, patients with pre-existing cardiovascular disease may suffer from worsened circulatory shock or cardiac arrhythmias when they experience significant blood loss or hypoxia. Similarly, older individuals with respiratory comorbidities are at increased risk of respiratory failure if they experience traumatic injuries to the chest, such as rib fractures or lung contusions. These comorbid conditions further complicate treatment and recovery, leading to higher mortality rates in elderly patients with multisystem trauma [12].

Another key factor that often contributes to the increased mortality rate in elderly patients with multisystem trauma is the delayed recognition and management of injuries. Elderly trauma patients with multiple organ system injuries were more likely to experience delayed diagnoses and were therefore at higher risk of poor outcomes and death [13]. 

## Conclusions

Farming remains one of the highest-risk professions for injury and mortality due to its associated occupational hazards. This case underscores the severity of these risks and highlights the heightened vigilance healthcare providers maintain when evaluating farmers in the emergency department. The term “code farmer” has emerged to reflect the concern that an apparently straightforward presentation in this population may mask more serious, insidious underlying conditions.
